# Selective Induced Altered Coccidians to Immunize and Prevent Enteritis

**DOI:** 10.1155/2016/3952534

**Published:** 2016-09-18

**Authors:** Helieh S. Oz

**Affiliations:** Department of Physiology and Internal Medicine, College of Medicine, University of Kentucky Medical Center, Lexington, KY, USA

## Abstract

Microbiomic flora in digestive tract is pivotal to the state of our health and disease. Antibiotics affect GI, control composition of microbiome, and shift equilibrium from health into disease status. Coccidiosis causes gastrointestinal inflammation. Antibiotic additives contaminate animal products and enter food chain, consumed by humans with possible allergic, antibiotic resistance and enigmatic side effects. Purposed study induced nonpathogenic, immunogenic organisms to protect against disease and abolish antibiotics' use in food animals and side effects in man. Diverse species of Coccidia were used as model. Immature organisms were treated with serial purification procedure prior to developmental stages to obtain altered strains. Chicks received oral gavage immunized with serial low doses of normal or altered organisms or sham treatment and were challenged with high infective normal organisms to compare pathogenicity and immunogenicity. Mature induced altered forms of* E. tenella *and* E. necatrix* lacked developmental stage of “sporocysts” and contained free sporozoites. In contrast,* E. maxima* progressed to normal forms or did not mature at all. Animals that received altered forms were considerably protected with higher weight gain and antibody titers against challenge infection compared to those that received normal organisms (*p* < 0.05). This is the first report to induce selected protective altered organisms for possible preventive measures to minimize antibiotic use in food animals.

## 1. Introduction

The microbiomic flora living in the digestive tract plays a pivotal role in the state of our health and disease. Recent investigations have been focused on medication use specifically antibiotics that affect digestive tract, which can control the microbiome composition and shift the equilibrium from health into disease status [1].

The recent decades are associated with the severe increases in the prevalence of IgE-mediated [2] and non-IgE-mediated food allergies and coincide with the rise in the antibiotic supplementation in poultry and livestock. For instance, the glioma development has been linked with elevated IgE and possible food allergies. Additionally, glioma patients have shown drastically higher rate of penicillin skin positive test with elevated blood eosinophil counts than the nonglioma control subjects [3]. These findings indicate an intricate association amongst glioma progression and possible state of combined food and antibiotic allergies in these patients.

The increase in antibiotic resistance has created a complex dilemma facing the public wellbeing. Indeed, the use of antibiotics against infections has created conundrum, as high doses may facilitate evolution of antibiotic resistance than curing infections. On the other hand, antibiotics' combination and extended period of treatments can promote severe injuries [4]. Further, suboptimal dose of antibiotics use as recently recommended may not act sufficiently enough to eradicate the pathogens.

Meanwhile, current consumers' awareness regarding the antibiotics residue contamination of poultry products and antibiotic resistant pathogenic microbial has caused concerns and recommendations to minimize the use of antibiotics as growth promoters in livestock in the USA [5] and worldwide.

Coccidiosis is a costly infectious disease which causes morbidity as well as fatality in livestock and poultry industry. So far, there is no safe and protective vaccine or practical prevention available [6]. Coccidians are host defined apicomplexan organisms which exclusively cause gastrointestinal inflammatory complications and diarrhea in man and animals. In contrast,* Toxoplasma*, another related member of Apicomplexa with intraintestinal (coccidian forms) and extraintestinal stages, is cosmopolitan and infects animals and man as well as every single organ and cell in the body [7]. Coccidiosis is one of the major diseases related to the food animals. Losses are directly due to morbidity as coccidiosis results in reduction in weight gain and egg production as well as affecting the quality of meat by decreased feed conversion, malabsorption, and maldigestion and further leads to mortality [8].* Eimeria* infection predisposes animals to clostridial infection and necrotic enteritis [9]. Common preventive practice includes the use of subtherapeutic doses of antibiotic additives into poultry and livestock diets which contaminate eggs, milk, and meat production. Antibiotics enter the food chain and are consumed by humans with possible allergic, antibiotic resistance [10] and other yet elusive side effects. For instance, the use of sulfonamide as anticoccidial has encountered the emergence of drug-resistant strains [11]. The estimated annual cost of coccidiosis in broiler production alone has been tripled ($350 million) in last decades in USA [12, 13] mainly due to anticoccidial drugs used to control the infection. Although continuous medication in feed has been effective against the disease outbreak, there are several disadvantages including sequential development of drug resistance and side effects in the consumers which necessitate constant search for new and effective compounds and vaccination. Animals that recover from the infection develop species specific immunity induced by the exposure to organisms or live vaccines. The viable vaccination with Coccivac, a mixture of diverse pathogenic species of birds'* Eimeria*, has been used for over 1/2 century in the United States [14]. This live vaccine causes (1) poor feed conversion, (2) a long duration required to evolve immunity against the disease, (3) possibility of introducing infection, (4) and difficulties of administering and managing the flocks. Other experimental vaccines are the attenuated strains including serial passages of* E. tenella* into chorioallantoic chick embryo [15] which were found to be ineffective. Since their discovery over a century ago [16],* Eimeria* have been described as organisms with four encapsulated sporocysts, each containing two infectious organisms called “sporozoites.”

The purpose of this study was to introduce nonpathogenic but immunogenic organisms to protect against the disease and to abolish the use of antibiotics in food animals and the side effects in the consumers. By utilizing purification procedure, altered* E. tenella* and* E. necatrix* were provoked to evolve 8 free sporozoites with no sporocyst formation. As a proof of concept, these altered organisms were less pathogenic than normal organisms but similarly immunogenic to normal forms. This investigation reports the formation of altered organisms to be serendipitous and to depend on the species of the organisms while their immunogenicity was reported to be comparable to normal organisms with use of birds as model and to warrant future investigations.

## 2. Materials and Methods

### 2.1. Ethics

This investigation was conducted according to the guidelines and approved by the Institutional Biosafety and the Institutional Animal Care and Use Committee (IACUC).

### 2.2. Animals

One-day-old specific pathogen-free (SPF) inbred White Leghorn chicks were obtained from the Poultry Science and kept in Coccidia-free and pathogen-free rooms, in daily disinfected wire-floor cages, and provided feed and water* ad libitum*. Each bird was tagged with a leg band for identification and weighed prior to, during, and at the end of the study. Weight gain/loss was calculated by subtracting the initial weight before inoculation from the weight obtained/lost following challenge and before termination of the study.

### 2.3. Oocysts Preparation

Sporulated oocysts of* E. tenella, E. necatrix,* and* E. maxima* were originally obtained from Eli Lilly and Company (Indianapolis, IN). Fresh cultures were prepared from the ceca or intestines of donor animals one week after oral inoculation. The contents were homogenized and the unsporulated organisms (oocysts) were separated from debris by sieve and centrifuged at 400 ×g and sediment added into 2.5% aqueous potassium dichromate (Sigma, St. Louis, MO) to obtain normal sporulated oocysts. In order to obtain altered oocysts, the homogenate containing unsporulated oocysts was further cleaned in a solution of 2.5% sodium hypochlorite and rinsed with distilled water (2x). The homogenates were centrifuged for 10 min at 400 ×g in a saturated salt solution (9 : 1 v.v) and then rinsed in distilled water. Oocysts were cultured in 2.5% aqueous potassium dichromate solution to enhance sporulation. The organisms were enumerated using a hemocytometer and diluted in phosphate buffer saline (PBS) according to each experiment before oral gavage directly into animal's crop. Normal control animals received sham (PBS) treatment.

### 2.4. Infection

At 2–6 weeks of age, the animals were inoculated via crop with 240, 2400, and 24000 sporulated oocysts of either the normal or altered strain one up to three doses according to the experimental design with a two-week interval. Infected birds were kept in isolation cages in a separate room and provided with food and water* ad lib*. One week after the challenge inoculation, animals were weighed and humanely euthanatized by cervical dislocation immediately followed by open chest and cardiac exposure. Different organs including segments of ceca and intestines were immediately removed and slides smears were prepared to detect the organisms. Cecal lesions were scored from 0 to +5 according to the severity of infection [8], briefly as follows:

 0 = normal mucosa and negative cecal/intestinal smears.
 1 = no detectable pathology but organisms detected in the smears.
 2 = scattered petechia on mucosa, organisms present on the smears, and normal cecal/intestinal contents.
 3 = focal inflammation and thickening of mucosa and isolated hemorrhage detected in lumen.
 4 = multifocal inflammation and thickening of mucosa and extensive hemorrhage in the lumen, with little or no fecal contents.
 5 = enlarged cecum/intestine with blood or sloughed off mucosa and moribund or dead animals.

### 2.5. Oocysts Sporulation

Organisms' growth and sporulation were determined via microscopic examination. Representative samples were examined and percentage of sporulated oocysts determined every 4 h. Sporulation was achieved when >90% of organisms had achieved sporulation.

### 2.6. Anti-*Eimeria* Antibody Detection by ELISA

The Enzyme Linked Immunosorbent Assay (ELISA) was performed using a soluble oocyst antigen of* E. tenella* according to [8, 17]. Blood was collected into syringe from the brachial vein from live animals and serum separated following 5 min centrifuge. Each serum sample was heat inactivated at 56°C for 30 min before use and diluted in twofold increments. Peroxides conjugated *γ* chain rabbit anti-chicken IgG (Cappel Laboratories, West Chester, PA) was dispensed into each microwell coated with 11 *μ*g of antigen and incubated with o-phenylenediamine (Eastman Kodak Co, Rochester, NY). Optical densities (OD) were documented at 490 nm on ELISA reader (DYnatech Lab Inc., Alexandria, VA).

### 2.7. Pathogenicity and Immunogenicity

*E. tenella* organisms were used as a model to compare the immunogenicity and pathogenicity of normal versus altered organisms. Oral gavage into crop with 240 and then 2400 oocysts of normal or altered organisms with a two-week interval was performed on three-week-old inbred White Leghorn chicks. Then animals were challenged with high infective (24,000) dose of normal* E. tenella*. One week after each inoculation, 6 animals per group were weighed and appearance and health status documented. Then blood samples from brachial vein were collected and animals humanely euthanatized by cervical dislocation and cardiac exposure followed by tissue specimens harvest.

### 2.8. Frozen Sections

Oocysts were centrifuged in 20% (W/V) bovine serum albumin (BSA) and 15% (W/V) sucrose. The pellets were fixed with 2 drops/mL of 25% glutaraldehyde. They were quick-frozen in liquid nitrogen and the blocks were sectioned at the 18 *μ*m setting on cryostat. Oocysts were embedded with mounting agent to protect the oocysts from shattering during the sectioning procedure. The sections were stained with Haematoxylin and Eosin.

### 2.9. Statistical Analysis

Results are expressed as mean ± SEM unless otherwise stated. Data were evaluated utilizing ANOVA followed by appropriate post hoc test. Statistical significance was set at *p* < 0.05.

## 3. Results

### 3.1. *In Vitro* Sporulation and Oocysts Description

**Figure 1:**
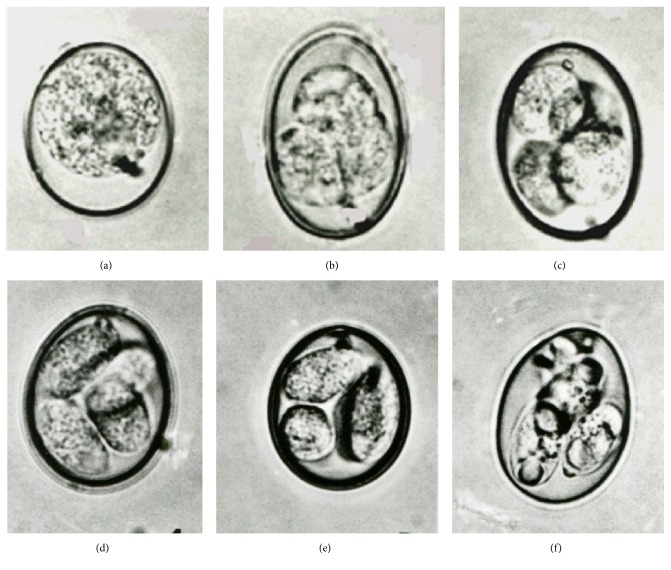
Normal oocysts of* Eimeria tenella* during* in vitro* growth and sporulation from 0 to 38 h (slide representative from over 100 organisms). (a) Immature organism (unsporulated oocyst) at 0 h × 1,000. (b) Normal organism start division at 16 h × 1,200. (c) Four spherical sporoblasts and a polar granule formation visible at 20 h × 1,100. (d) Ellipsoidal sporoblasts at 24 h × 1,000. (e) Sporocysts formation at 28 h × 1,000. (f) Sporocysts formation completed, each containing 2 mature sporozoites at 38 h × 1,000.

**Figure 2:**
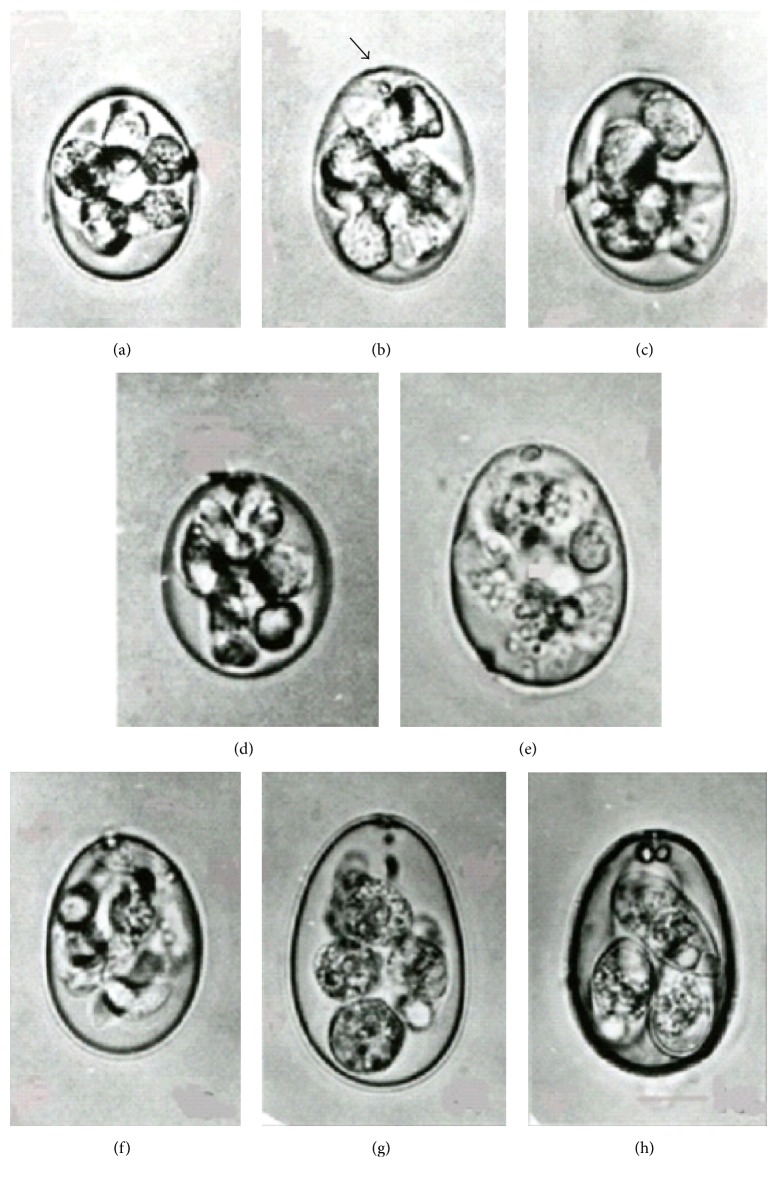
(a–e) Altered organisms (oocysts of* E*.* tenella*) during evolutional stages following procedure at 28 to 44 h (slide representative from over 100 organisms). (a) Altered organisms (oocysts) begin to divide at 28 h × 1,000. (b) Further development showing the polar granule (indicated by arrow) at 32 h × 1,200. (c) Free sporozoites forming from protoplasmic mass at 36 h × 1000. (d) Sporozoites formation complete at 44 h × 1,100. (e) Degenerating altered oocyst of* E*.* tenella* 3 months after storage at 4°C × 1,070. (f–h)* Eimeria necatrix* and* E*.* maxima* following the alteration procedure (slide representative from over 100 organisms). (f)* E*.* necatrix* proceeds to altered organism after the procedure to contain free sporozoites × 1,000. (g)* E*.* maxima* fail to complete sporulation after the altering procedure. × 800. (h)* E*.* maxima* form normal organisms containing 4 sporocysts, each with 2 sporozoites after the procedure. × 800.

Normal oocysts of* E. tenella* began cell division and sporulation 16–20 h in the external environment to each form of four internal sporoblasts. After 28 h four sporocysts were matured and the sporozoites' development was completed in 38 h (Figures 1(a)–1(f)). In contrast, altered oocysts initiated division approximately 12 h later than normal oocysts (28 h). Paradoxically, organisms started to flourish into newly formed free sporozoites at 36 h to shape no prior sporocysts formation and sporulation was completed after 44 h (Figures 2(a)–2(d)), that is, total 6 h longer than expected with normal oocysts. Greater than 95% of processed organisms developed into the altered form. The altered organisms were round to oval in shape (25 to 35 *μ*m by 22.5 to 27.5 *μ*m) and each contained eight banana-shaped free sporozoites (12.5–14 *μ*m by 2.5 *μ*m). While the measurements were in consistency with those of normal form, the exception was lack of sporocysts.

### 3.2. Effect of Storage on the Oocysts Lifespan

The altered organisms when stored at 4°C for 3 months; the free sporozoites within the oocysts then started to degenerate (Figure 2(e)). In contrast, normal oocysts preserved the appearance of sporocysts and sporozoites with no visible signs of degeneration over 1 year after storage at 4°C. This establishes the specific protective entity of the sporocysts provided for the longevity and infectivity of the sporozoites.

### 3.3. Altered Oocysts of Other Species of* Eimeria*

Attempts were made to determine whether or not parallel developmental alteration would occur with other related coccidian organisms. Similarly, the altered oocysts of* E. necatrix,* another pathogenic Coccidia, were induced utilizing the same methodology as described for* E. tenella* organisms. Likewise, altered* E. necatrix* oocysts contained eight free forming sporozoites and lacked sporocystic structures (Figure 2(f)). In contrast, when another species,* E. maxima*, was treated with similar procedure, the organisms either did not complete sporulation process or simply transformed into normal sporulated oocysts (Figures 2(g) and 2(h)).

### 3.4. Oocysts and Immunity

In order to investigate the immunogenicity of the organisms, the inbred animals each were given escalating doses from low 240 (Table 1) and then 2400 (Table 2) of normal or altered organisms ones with a two-week interval. The animals which were treated with altered strain all profoundly gained more weight (*p* < 0.05) and considerably showed higher antibody titers in sera compared to those immunized with normal organisms (*p* < 0.05). Two weeks after the last immunizing dose (2400 normal or altered forms), remaining animals were all challenged with high dose of 24,000 normal organisms (Table 3). As a result, those animals that received only the challenge dose (24,000 normal oocysts) drastically lost weight (*p* < 0.01) and developed bloody diarrhea compared to immunized ones. The pathological lesions were scored as 3.5 ± 0.5 with moderately severe inflamed and thickened intestinal mucosal and hemorrhage detected in mucosa and lumen in contrast to those immunized animals (*p* < 0.01, Table 3).

All uninfected animals as expected were sera antibody negative. Both immunized groups with either altered or normal forms showed no pathological lesions and were antibody positive with higher titers developed in those immunized with altered compared to normal organisms. The major difference between inoculated groups was expressed with those animals immunized with altered organisms to gain considerably more weight and to be in better overall health status than those animals previously immunized with normal organisms (Table 3, *p* < 0.05).

## 4. Discussion

A major role for the vast population of intestinal microbiota is to prevent incursion and colonization of infectious microbial pathogens [1–18]. Antibiotics including the short term use can damage propagation of the commensal microbiome to favor susceptibility to dysbiosis. The commensal microbiota seems to be most important when established early in life [19]. Therefore, previous criteria such as cesarean versus natural birth and breastfeeding may no longer be considered as the main focal point. Therefore, the attention has been focused on the antibiotics which can control the microbiome composition [19] and the equilibrium between health and disease.

Noncommunicable and inflammatory complications such as allergic diseases affect over millions of people. The food allergies in westernized societies have been drastically increased mainly due to lifestyle and diet. Thus far, the pathogenesis of allergies specifically food related allergies has remained somehow elusive [20]. This coincides with the upsurge in the prevalence of IgE-mediated food allergies unsolved in children yet with possible environmental [2] and/or nutritional exposures involvement. Additionally, a profound relationship has been detected between the use of macrolide antibiotics and asthma development amongst European children (6–36 months of age) as in a multicenter prospective trial. There was a definite correlation between the use of macrolide in the first year of life and wheezing in 3-year-olds, independent of the respiratory infections [21]. Meanwhile, high prevalence of fluoroquinolone and macrolide resistance pathogens including* Campylobacter* in poultry and swine industry [22] due to misuse raises alarm about possible overexposure and ineffectiveness of these antibiotics in humans' therapy.

A link between exposures to environmental factors has been demonstrated in development of chronic inflammatory diseases such as Crohn's and ulcerative disease. These revelations comprise childhood exposure to different dietary elements [23]. Paradigm includes food protein induced enterocolitis syndrome (FPIES) as a non-IgE-mediated hypersensitivity in children that provokes symptoms of severe vomiting, diarrhea, and acidosis a few hours following consumption of dietary proteins, chicken, turkey, egg white, and cow's milk [24]. Indeed, eggs are reported as a frequent trigger of FPIES [25]. In sporadic cases in infants, the non-IgE-mediated hypersensitivity has been noted to shift to typical IgE-mediated cow's milk allergy [26]. The T cell activation may or may not be present in FPIES [27], while the mechanism(s) which predispose(s) one to FPIES has remained an enigma.

Antibiotic use has a rapid and major effect on the luminal bacterial community followed by long-lasting consequences for regulation of microbial population architecture and the host immunity [28]. Recent studies stress the mucosal-associated commensal bacterial to form a protective barrier to prevent food allergic sensitization.* In vivo* and* in vitro* studies support utilization of oral live biotherapies (e.g., prebiotics) to modulate luminal microbiome and to induce tolerance against food allergy in patients [28].

In a retrospective case-control trial (2004–2013), patients with glioma (*n* = 913) had profoundly higher rate of penicillin skin positive (IgE) test and elevated blood eosinophil counts than matched controls (*n* = 1091) with a possible link between glioma and food allergies [3]. Yet, *β*-lactam antibiotics, penicillin (e.g., amoxicillin and dicloxacillin), and cephalosporins (e.g., cefazolin and cefotaxime) are regularly used in animals. Abuse and illegal use of these antibiotics [29] as well as macrolides have been detected in food animal products which can result in bacterial resistance and allergy. For instance, tetracycline, one of the most common antibiotics used in poultry, is deposited and remains in bones and is passed into food chain with potential human health risk regardless of enforced monitoring of proper withdrawal times [30]. Diclazuril is another antimicrobial routinely used in poultry industry to protect against coccidial infection. We have recently reported diclazuril monotherapy or combined with atovaquone to be effective against congenital and maternal toxoplasmosis [7, 31, 32]. The combination antibiotic therapy most effectively protected pups against severe multiorgan inflammatory reaction and fatality and to candidate diclazuril mono or combined therapy as an attractive antitoxoplasmosis in humans.

The antibiotic resistant pathogens are caused by the extensive utilization of subtherapeutic antibiotics feed to poultry [5] and livestock. Recent consumer awareness regarding the antibiotics residual contamination in poultry products and the antibiotic resistant pathogenic microbial has created great concerns and recommendations to minimize the use of antibiotics as growth promoters in livestock in the USA [5]. Alternative preventive measures are sought, desirable to eliminate or minimize the use of antibiotics in livestock and poultry industry.

In this study, the altered organisms differed from normal oocysts in that they lacked sporocysts, even though each contained eight free sporozoites. The altered organisms produced considerably less pathogenicity in immunodeficient animals compared to normal oocysts (unpublished data), while the protection induced by these organisms in immunocompetent inbred animals was no less than those induced by normal forms. Indeed, animals inoculated with altered organisms gained more weight and demonstrated minimal pathological symptoms due to infection and induced higher antibody titers compared to those that received normal forms. One century after the original discovery of eimerians, a reproducible method of altering infective forms to noninfective ones has been introduced here. Previous studies have used irradiation to alter the pathogenicity of the organisms [33, 34] or to induce resistance by the genetic selection of the avian host [35]. The intramuscular injection of the plasmid pcDNA3-1E of* E*.* acervulina* was reported to be safe in chicken [36]. Also,* E*.* tenella* EtMic2 (a microneme protein) displayed on the cell surface of* S*.* cerevisiae* used orally as a live vaccine was reported to protect against challenge infection by lowering lesion scores and fecal oocysts in birds and stimulated humeral innate immunity [37]. Our interrogation indicated that the process of mechanochemical alteration in developmental stages prior to sporulation can rationally lead to formation of altered organisms (*E*.* tenella *and* E*.* necatrix*) with ability to immunize against severe infections and to protect against luminal inflammation.

Normal organisms are reported to preserve internal structure for up to 25 years in 2.5% potassium dichromate [38]. The current study proves that sporocysts have a protective effect on preserving infectivity and longevity, as altered organisms which lack sporocysts and their structural entity degenerate after only 3 months. The three diverse species of* Eimeria* tested in this investigation (*E*.* tenella*,* E*.* necatrix*, and* E*.* maxima*) all did not react in a similar manner to the altering procedure. While* E*.* tenella* and* E*.* necatrix* both similarly followed procedure for formation of abnormal organisms,* E*.* maxima* failed to be processed to altered formation with no obvious explanation for this variability. There are some discrepancies in the life cycles of these species.* E*.* maxima* morphologically are somewhat larger than either of the two species. However, this may possibly have little or no effect on the developmental behavior divergence observed here.

Overall, this report may inform the medical community of the vast unwanted antibiotics use and residues in animal products and a possible advancement in prevention of coccidiosis by other approaches than the use of antibiotics, therefore lessening the chances in consumption of antibiotics contaminated animal products and changes in luminal microbiota and prevalence of the food allergies consequences in humans.

## 5. Conclusion

This is the first report to induce selected protective altered coccidian organisms for possible preventive measures to minimize antibiotic use in food animals. The altered organisms and the subsequent protective immunity they produce against severe infection require further investigation.

## Figures and Tables

**Table 1:** Six White Leghorn birds/group were inoculated with normal (N) or altered (A) oocysts of *E. tenella *and euthanatized 1 week after. Control (C) birds received sham inoculation (0). Body weight gain/g.

Group	Strain	Oocyst/	Weight gain	Lesion	Ab titer (OD)
1	A	240	104 ± 5	1	0
2	N	240	85 ± 4	1	0
3	C	0	142 ± 8	0	0

**Table 2:** Six White Leghorn birds/group were immunized with 240 followed by 2400 normal (N) or altered (A) oocysts of *E. tenella* with a two-week interval (week 3). Control (C) birds that received sham inoculation (0) gained most but with no titers. Body weight gain/g and sera antibody titer production in group A versus group N showed significant difference; ^*∗*^ *p* < 0.05.

Group	Strain	Oocyst/	Weight gain	Lesion	Ab titer (OD)
4	A	2400	95 ± 4^*∗*^	1	42.0^*∗*^
5	N	2400	66 ± 6	1	25.0
6	C	0	180 ± 9	0	0

**Table 3:** White Leghorns were immunized with 240 and 2400 normal (N) or altered (A) oocysts of *E. tenella* with a two-week interval. Infected control group (N^∧^) received no previous immunization before final challenge. Remaining birds were challenged each with 24,000 normal oocysts. Controls (C) received (0) sham inoculation. N^∧^ lost weight the most and developed moderately severe lesions. Body weight gain/g was significantly higher in uninfected animals and those immunized with altered compared with the normal organisms and there was weight loss in unimmunized animals. N = 6 animals/group. ^#^ *p* < 0.01, ^*∗*^ *p* < 0.05. ND = not done.

Group	Strain	Oocyst/	Weight gain	Lesion	Ab titer (OD)
7	A	24,000	71 ± 4^*∗*^	0	50.0
8	N	24,000	21 ± 4	0	38.0
9	N^∧^	24,000	−50^#^	3.5 ± 0.5^#^	ND
10	C	0	171 ± 4	0	0
